# Supplementary material for: Massive-scale single-nucleus multi-omics identifies novel rare noncoding drivers of Parkinson’s disease
Source: bioRxiv. 2026 Mar 5:2026.03.05.709922. Preprint. [Version 1] doi: 10.64898/2026.03.05.709922 (PMC13001318; doi:10.64898/2026.03.05.709922)
Supplement: 1 [file NIHPP2026.03.05.709922V1-supplement-1.pdf]

bioRxiv preprint doi: https://doi.org/10.64898/2026.03.05.709922; this version posted March 5, 2026. The copyright holder for this preprint (which was not certified by peer review) is the author/funder, who has granted bioRxiv a license to display the preprint in perpetuity. It is made available under aCC-BY 4.0 International license.

Supplementary Figure 1. Multi-omic profiling of postmortem human brain yields high-quality and high-depth data across 101 donors.

a. Schematic of workflow for snATAC-seq and snRNA-seq processing from pooled postmortem brain nuclei. Nuclei from multiple donors were pooled across 642 10x Genomics Multiome reactions. Reads were aligned, donors were demultiplexed, and doublets were removed separately per modality. Modality-specific quality control filters were then applied: snATAC-seq (TSS enrichment > 3.5, nFrags ≥ 1,000, with manual nFrags thresholding), and snRNA-seq (nGenes > 100, mitochondrial reads < 10%). Final datasets: snATAC-only (4.2M nuclei), snRNA-only (4.5 M nuclei), and multi-omic (3.3 M nuclei).

b. Strip chart displaying donor distributions of age at death and biological sex across the Unified Staging System for Lewy Body Disorders. Each point is a donor, colored by biological sex (blue – male, pink – female).

c. Matrix plot showing donor-level metadata. Columns show donors; rows include disease status, biological sex, Unified Lewy Body (LB) Stage, age at death, postmortem interval, years since diagnosis, clinical/neuropathology metrics, and quantitative region-specific pathology scores.

d. Batch-level quality control for each modality. Box and whisker plots show (left to right within each modality): $\log_{10}$(unique fragments) (ATAC-seq; orange), TSS enrichment score (ATAC-seq; blue), number of detected genes (RNA; green), number of UMIs (RNA; red), and mitochondrial read percentage (RNA; purple). Each row corresponds to a processing batch. The center line denotes the median, the box spans the interquartile range (IQR; 25th–75th percentiles), and the whiskers extend to the most extreme data points within 1.5× the IQR. Outliers are not shown for cleaner visualization.

e. Stacked bar plot displaying fraction of nuclei passing modality-specific quality control per brain region.

f. Scatter plot of per-cell snATAC-seq TSS enrichment versus snRNA-seq genes per UMI for multi-omic nuclei; linear fit shown with Pearson correlation coefficient (r) and *p*-value from Pearson correlation test.

g. Box and whisker plot comparing snATAC-seq TSS enrichment for nuclei freshly isolated from frozen tissue compared to nuclei isolated, cryopreserved, and then thawed across regions. The center line denotes the median, the box spans the interquartile range (IQR; 25th–75th percentiles), and the whiskers extend to the most extreme data points within 1.5× the IQR.

Supplementary Figure 2. Sample multiplexing and genetic deconvolution enable uniform profiling across samples without observable batch effects across key covariates.

a. Bar plot of the number of nuclei passing filtering thresholds per sample across each brain region. The bottom panel displays the combined totals across all regions for all 101 donors. Each bar represents one donor and is colored by disease status.

b–g. UMAP embedding of nuclei colored by (**b**) broad cell type, (**c**) brain region of origin, (**d**) biological sex, (**e**) disease status, (**f**) donor identity, and (**g**) sequencing batch.

bioRxiv preprint doi: https://doi.org/10.64898/2026.03.05.709922; this version posted March 5, 2026. The copyright holder for this preprint (which was not certified by peer review) is the author/funder, who has granted bioRxiv a license to display the preprint in perpetuity. It is made available under aCC-BY 4.0 International license.

Supplementary Figure 3. Cell type annotation identifies differences in brain regional composition and data quality across cell subtypes.
a. Bar plot of the total number of nuclei per cell type across the dataset. Colors denote the brain region of origin.
b. Bar plot of proportion of cell subtypes within each brain region.
c. Stacked bar plot showing the proportion of cell subtypes contributed by each donor. Each column represents one donor.
d. Quality control metrics displayed by cell subtype. In order from left to right: stacked bar plot showing the percent of nuclei passing modality-specific filters (ATAC-only pass, RNA-only pass, or passing both, violin plots of snATAC-seq metrics, including the number of fragments (nFrags) and TSS enrichment score, and violin plots of snRNA-seq metrics including number of detected genes (nGenes), number of UMIs (nUMI), and $\log_{10}$(genes per UMI).

Supplementary Figure 4. Subclustering of dopaminergic and putamen neuron populations uncovers subtypes of neurons with distinct molecular signatures.
a. UMAP embedding of the RNA modality showing dopaminergic neurons, colored by subcluster identity.
b. Dot plot showing subcluster representation across dopaminergic neuron modules. Point size indicates the percentage of cells expressing each module, and dot color encodes the average module eigengene value across clusters. Right panel: GO term pathway enrichment results for each module.
c. UMAP embedding of (**c**) all putamen nuclei (RNA modality), colored by refined cell subtype, (**d**) putamen neurons (RNA modality), colored by refined neuronal subtype, and (**e**) putamen neurons (ATAC modality), colored by refined neuronal subtype.
f. Dot plot of selected marker genes across putamen neuronal subtypes. Point size indicates the percentage of cells expressing each gene and point color indicates the z-scored average log-normalized expression level of each gene across all cells in that class.

Supplementary Figure 5. Subclustering and marker gene expression identify regional differences in non-neuronal cell populations.
a. UMAP embeddings of astrocytes, capillary endothelial cells, microglia, oligodendrocytes, and oligodendrocyte precursor cells (OPCs) colored by (left) brain region and (right) subcluster identity.
b. Cluster residence heatmaps showing the proportion of cells in each subcluster originating from each brain region, colored by percent of cells residing in each subcluster.
c. Heatmaps of selected marker gene expression across subclusters for each glial population. Values are shown as z-score-normalized expression per gene.

Supplementary Figure 6. Differential gene expression analysis combined with permutation testing highlight cell types with statistically significant levels of molecular alteration between PD and control individuals.
a. Heatmap displaying Pearson correlation coefficients between donor-level covariates (age at death, biological sex, postmortem interval (PMI)) and principal components (PC1–PC5) computed from pseudobulk expression profiles for each cell type. Values are shown per cell type/principal component. Principal components without significant correlation with covariates are excluded from the plot.
b. Density plots showing the distribution of the number of significant differentially expressed genes (DEGs; $\log_{10}$-transformed) across 10,000 permutations of case/control label shuffling for each cell type. Vertical dashed lines indicate the observed number of DEGs in the real dataset. Reported *p*-values represent the proportion of permutations with equal or greater numbers of

bioRxiv preprint doi: https://doi.org/10.64898/2026.03.05.709922; this version posted March 5, 2026. The copyright holder for this preprint (which was not certified by peer review) is the author/funder, who has granted bioRxiv a license to display the preprint in perpetuity. It is made available under aCC-BY 4.0 International license.

DEGs than observed; N indicates the number of donors for that cell type. Labels in green indicate a significant number of observed DEGs ($p < 0.05$); all other labels in red.

c. Heatmap comparing $\log_2$(fold change) values of the top 2 DEGs by fold change across cell types. Rows represent cell types and columns represent genes. Color scale reflects the $\log_2$(fold change) of PD compared to Control. Asterisks indicate genes that are significant (FDR < 0.05) in the corresponding cell type based on a likelihood ratio test.

d. Scatterplot comparing the median number of cells per donor and the number of DEGs identified as significant across the 29 tested cell types. Point color represents cell type as used throughout the manuscript. Fit line represents linear regression with the 95% confidence interval shown in gray.

Supplementary Figure 7. Differential gene expression analysis uncovers shared molecular signatures across brain regions.

a. Dot plot of pathway enrichment of DEGs in Dopaminergic Neurons (left) and Excitatory Neurons (right). Point size represents the gene count per pathway; color represents the $-\log_{10}$(p-value) of enrichment.

b. Histogram displaying permutation-based background distributions for the number of shared DEGs between pairs of brain regions in cell types with DEGs observed in multiple regions (Astrocytes, Excitatory Neurons, Oligodendrocytes). Dashed lines indicate the observed number of shared DEGs.

c. UpSet plot showing overlap of DEGs across brain regions for Oligodendrocytes. Intersection sizes are shown on the left; the inset bar plot displays the total number of DEGs identified in each region.

d Heatmap of selected marker genes displaying region-specific differential expression across cell types. Colors represent $\log_2$(fold change) of PD compared to Control; asterisks indicate significance (FDR < 0.05) based on a likelihood ratio test.

e. Dot plot of enrichment of the overlap of region-specific DEGs within Oligodendrocyte subcluster DEGs. Point size represents the number of overlapping DEGs from each region and each subcluster, and color corresponds to the $-\log_{10}$ (FDR) of the enrichment of the overlap of genes within the two groups. Gray color indicates not significant.

f. Dot plot of pathway enrichment of DEGs shared between brain region pairs in Oligodendrocytes. Point size represents the number of overlapping genes; color indicates $-\log_{10}$ (FDR) of the enrichment of overlapped genes within the given pathway. Gray color indicates not significant.

Supplementary Figure 8. Cell type proportions and gene expression show distinct patterns across the trajectory of Lewy body accumulation in PD.

a. Box and whisker plot showing log-transformed cell type proportions in control and PD donors. Each column represents a different cell type. Outlier donors are indicated as points. For box and whisker plots (**a,c,d**), the center line denotes the median, the box spans the interquartile range (IQR; 25th–75th percentiles), and the whiskers extend to the most extreme data points within 1.5× the IQR.

b. Heatmap of the $\log_2$ (PD / Control) ratios of cell type proportions across the brain regions containing cell types that showed differential abundance in **Figure 2c**.

c. Box and whisker plots showing cell type proportions across stages of the Unified Staging System for Lewy Body Disorders for interneuron and glial populations with significant difference in observed cell type proportions between PD and Control individuals (Chandelier, LAMP5, Oligodendrocytes, SST, VIP). Linear trend lines are shown with p-values and R^2 values from linear regression.

d. Box and whisker plots of chromatin accessibility signal in Astrocytes at peaks mapping to *PRLR*, shown across stages of the Unified Staging System for Lewy Body Disorders. Each point

bioRxiv preprint doi: https://doi.org/10.64898/2026.03.05.709922; this version posted March 5, 2026. The copyright holder for this preprint (which was not certified by peer review) is the author/funder, who has granted bioRxiv a license to display the preprint in perpetuity. It is made available under aCC-BY 4.0 International license.

represents a donor, and FDR values indicate the significance of stage association using likelihood ratio tests, which evaluate whether accessibility varies with Lewy body stage while adjusting for disease status.
e. Plot of the mean $\log_2$ expression trajectories across stages of the Unified Staging System for Lewy Body Disorders for oligodendrocyte genes grouped by monotonic or late-stage expression patterns. Shaded regions represent the standard deviation.
f. Left: Dot plot showing the proportion of genes in each cell type assigned to each temporal expression pattern category (late up, monotonic up, late down, monotonic down). Point size reflects the observed proportion; shading indicates deviation from the expected proportion. Right: Stacked bar plot showing the percentage of genes in each pattern category by cell type.

Supplementary Figure 9. Cell type-specific ChromBPNet ML models learn chromatin accessibility patterns with comparable accuracy across cell types.
a. Bar plot indicating variant effect size thresholds per cell type. Positive bars (red) indicate the threshold of positive effect (increased accessibility), and negative bars (blue) indicate the threshold of negative effect (decreased accessibility).
b. Scatter plot of the average Pearson correlation of ML model predicted counts with observed counts across cross-validation folds plotted against the number of cells used for training, per cell type. Points are colored by cell type. Point size indicates the average median normalized Jensen–Shannon divergence (norm JSD) between observed and predicted accessibility profiles across regions. JSD values were normalized relative to a worst-case uniform prediction and a best-case perfect prediction (JSD = 0), yielding values between 0 and 1; larger points indicate closer agreement between predicted and observed profiles. This is averaged across 5 training folds.

Supplementary Figure 10. Cell type-specific Micro-C identifies variant-to-gene interactions.
a–b. Representative fluorescence-activated nuclei sorting scatter plots of (**a**) the sorting strategy used and (**b**) the post-sort purity of the nuclei used for downstream Micro-C library generation.
c. Schematic overview of Micro-C dataset generation. Cell type-specific nuclear markers used for sorting are indicated beneath each cartoon.
d. Bar plot comparing total unique loops identified in Micro-C libraries generated from control samples and PD samples across astrocytes, microglia, neurons, and oligodendrocytes. Loop sizes are stratified by genomic span.
e. Line plot showing the number of loops per 10-kb bin resolution, across astrocytes, microglia, neurons, and oligodendrocytes.
f. Density plots of the distribution of genomic distances between loop anchors, across cell types.
g. Bar plots of genomic annotations of loop anchor combinations per cell type.
h. Stacked bar plot displaying the proportion of loop anchors overlapping accessible chromatin peaks in each cell type.
i–l. Representative chromatin contact matrices for each cell type profiled, with contact intensities visualized as normalized Micro-C signal.

Supplementary Figure 11. Multi-omic mapping links rare noncoding variants to precise, cell type-specific target genes.
a. Overview of the variant-to-gene mapping workflow. Numbers shown refer to combinations of variants and cell types as some individual variants are assessed across multiple cell types.
b. Stacked bar plot showing the number of rare variants (MAF < 0.01) identified as “hit variants” per cell type across the dataset. Stacked colors represent the mapping strategy used to assign the variant to its putative target gene.

bioRxiv preprint doi: https://doi.org/10.64898/2026.03.05.709922; this version posted March 5, 2026. The copyright holder for this preprint (which was not certified by peer review) is the author/funder, who has granted bioRxiv a license to display the preprint in perpetuity. It is made available under aCC-BY 4.0 International license.

c. Density distributions of variant-to-TSS distances for each mapping strategy. The red vertical line denotes the median nearest-TSS distance.
d. Stacked bar plot showing the number of variants linked to nearest versus non-nearest genes for each mapping method.
e. Bar plot of the top 20 genes with the highest counts of predicted effect variants normalized by genomic area mapped to each gene per cell type.
f. Distribution of cell type specificity (Tau) values for genes targeted by rare noncoding variant hits. Observed data (blue) and permuted background distributions (gray) are shown. The red dashed line marks the 95th percentile of the permuted distribution (Tau = 0.67), used as the significance cutoff.
g. Histogram showing the number of cell types in which each gene is found to be enriched for rare noncoding variants.
h. Pathway enrichment analysis of genes enriched for rare noncoding variant hits. Bars represent significant Gene Ontology terms, colored by *p*-value.
i. Normalized pseudobulked chromatin accessibility tracks for the genomic region surrounding *CSMD1*. The area immediately surrounding variant chr8:3335995 – G to A is highlighted in light blue.
j. Importance score plots for the variant shown in (**h**), including reference, alternate, and delta signal, with the associated JUNB TF motif shown below. Importance score ranges for each plot are shown in parentheses.

Supplemental Figure 12. Rare variant association testing in PD identifies genes and putative regulatory elements enriched for noncoding variants.
a. Quantile–quantile (QQ) plot of observed versus expected $-\log_{10}$(*p*-values) from a representative cell type-specific rare variant association test (shown for Oligodendrocytes).
b. Dot plot of the number of significant rare variant-associated features per cell type. Point size reflects the number of significant features, and point color indicates whether the feature corresponds to a gene-set or a chromatin accessibility peak.
c. Manhattan plot of rare variant association testing in the UK Biobank replication cohort of loci discovered in the GP2 cohort. The dashed red line denotes the replication significance threshold. Points are colored by cell type.
d. Box and whisker plot showing log2(CPM+1) expression of *LRFN2* across stages of the Unified Staging System for Lewy Body Disorders. Pairwise stage differences were assessed using two-sample t-tests with pooled variance from a one-way ANOVA model; raw (unadjusted) *p*-values are shown.
e. Manhattan plot of peak-based rare variant association testing with cell type-significant peaks colored by cell subtype. Labeled variants represent associations that are globally significant. Gray points indicate peaks that were not significant when tested in any individual cell type.

Supplementary Tables and Data

Supplementary Table 1. Cohort Metadata
Donor-level metadata for all 101 individual donors.

Supplementary Table 2. Differential gene expression results
Per cell type differential gene expression results.

Supplementary Table 3. Pathway enrichment for differential gene expression results
Per cell type pathway enrichment of differentially expressed genes.

bioRxiv preprint doi: https://doi.org/10.64898/2026.03.05.709922; this version posted March 5, 2026. The copyright holder for this preprint (which was not certified by peer review) is the author/funder, who has granted bioRxiv a license to display the preprint in perpetuity. It is made available under aCC-BY 4.0 International license.

Supplementary Table 4. Differential chromatin accessibility results
Differential accessibility analysis of peaks linked to differentially expressed genes.

Supplementary Table 5. DEG Trajectory results
Trajectory analysis across PD pathological progression for all DEGs.

Supplementary Table 6. Pathway enrichment of trajectory results
Pathway enrichment for each identified trajectory of DEGs across the PD pathological progression.

Supplementary Table 7. Gene-level RVAT results
RVAT results for gene-level aggregation of noncoding rare variants.

Supplementary Table 8. Peak-level RVAT results
RVAT results for peak-level aggregation of noncoding rare variants.

Supplementary Table 9. Familial PD variants prioritized by ChromBPNet models
All noncoding variants prioritized by ChromBPNet models from Family 1 and Family 2.

Supplementary Table 10. GP2 banner author list
The full list of authors that contributed to this manuscript as part of the Global Parkinson's Genetics Program.

Supplementary Table 11. snMultiome data availability
All accession and download information for publicly available raw and processed snMultiome data, hosted on the Impact of Genomic Variation on Function (IGVF) consortium data portal.

Supplementary Data 1. Expression and chromatin accessibility QTL results
All relevant results for eQTL and caQTL calling are presented.

Supplementary Data 2. ML-prioritized rare noncoding variants
All ML results for rare noncoding variants prioritized from sporadic PD cases are presented.

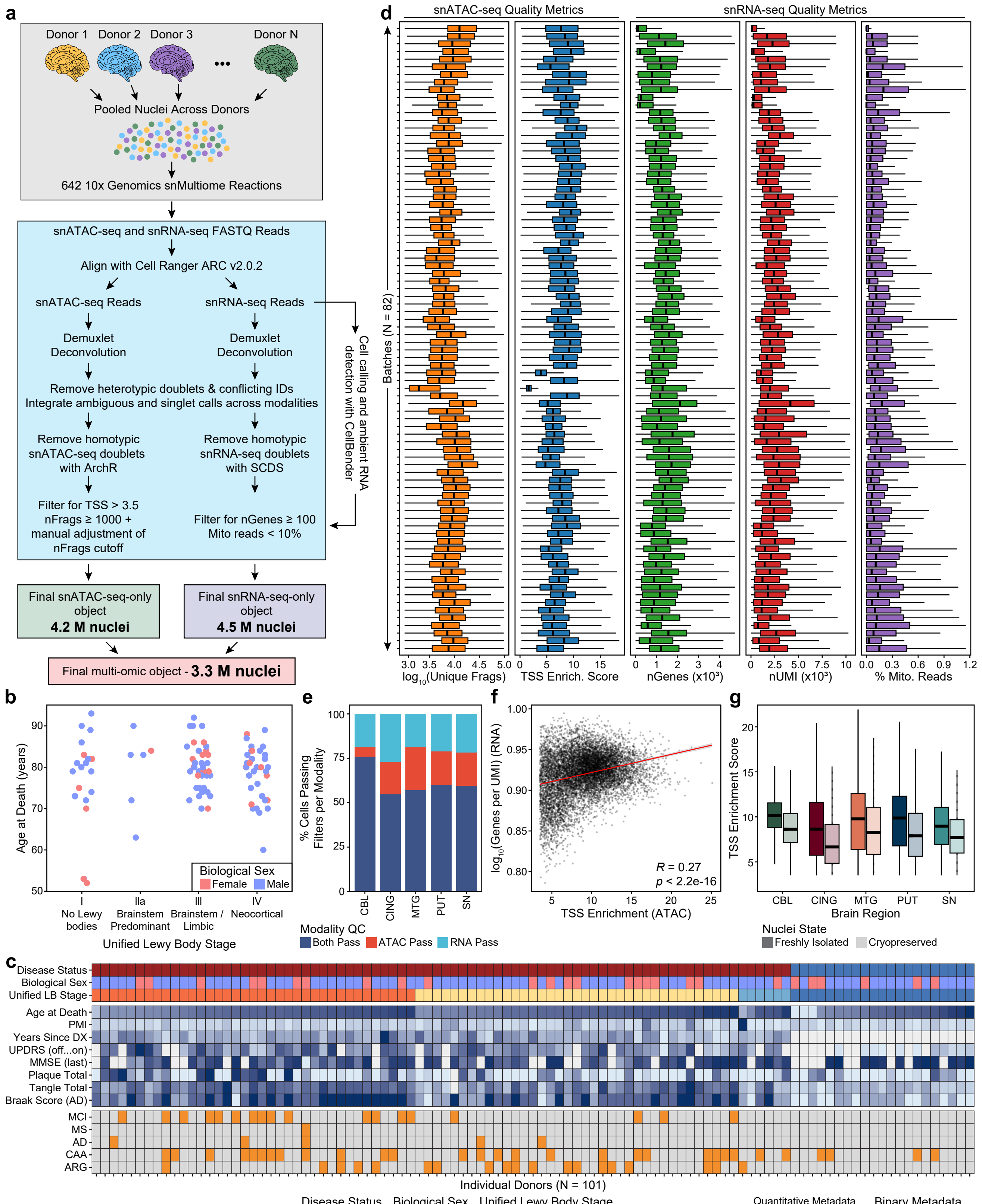

Supplementary Figure 1

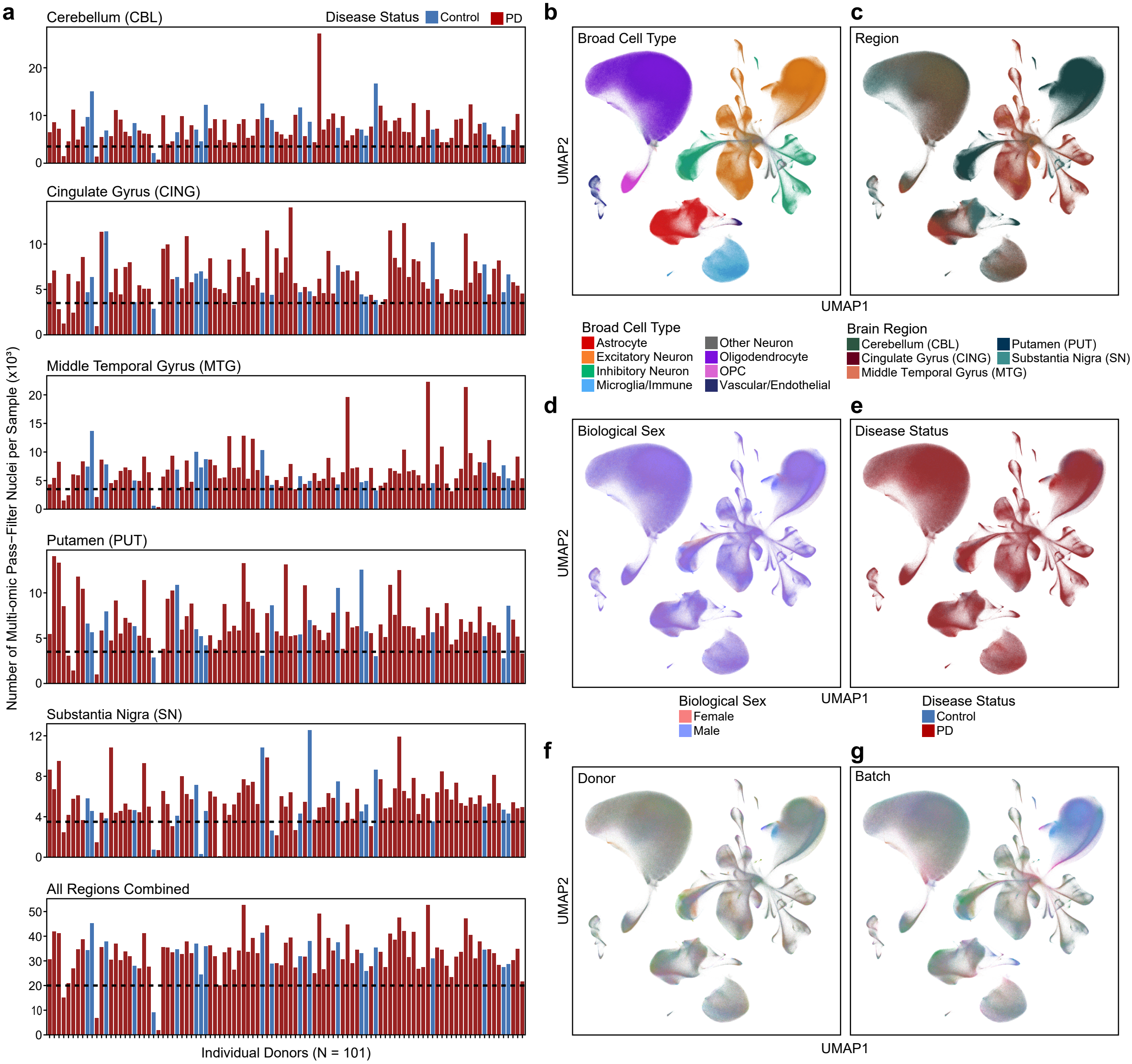

Supplementary Figure 2

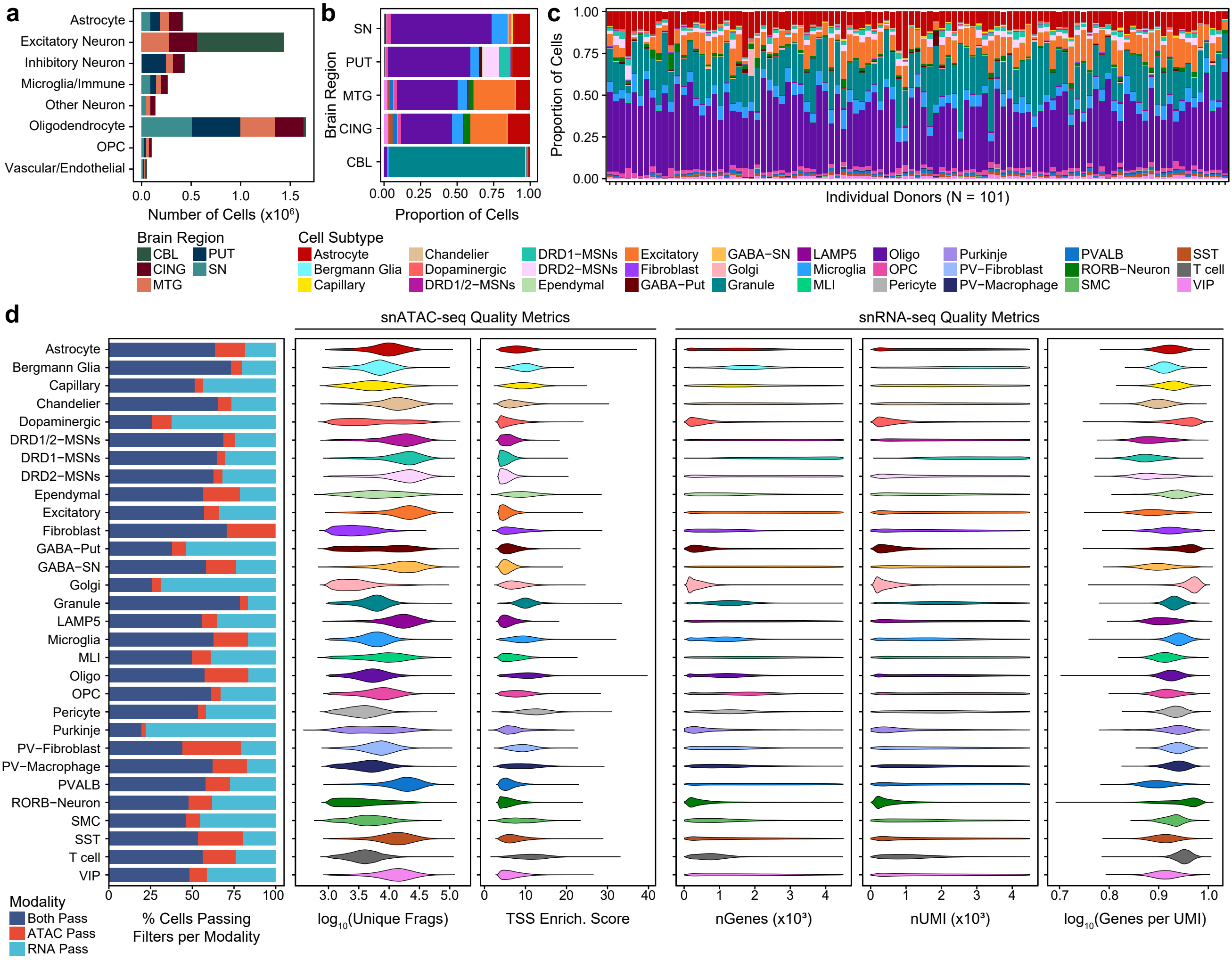

Supplementary Figure 3

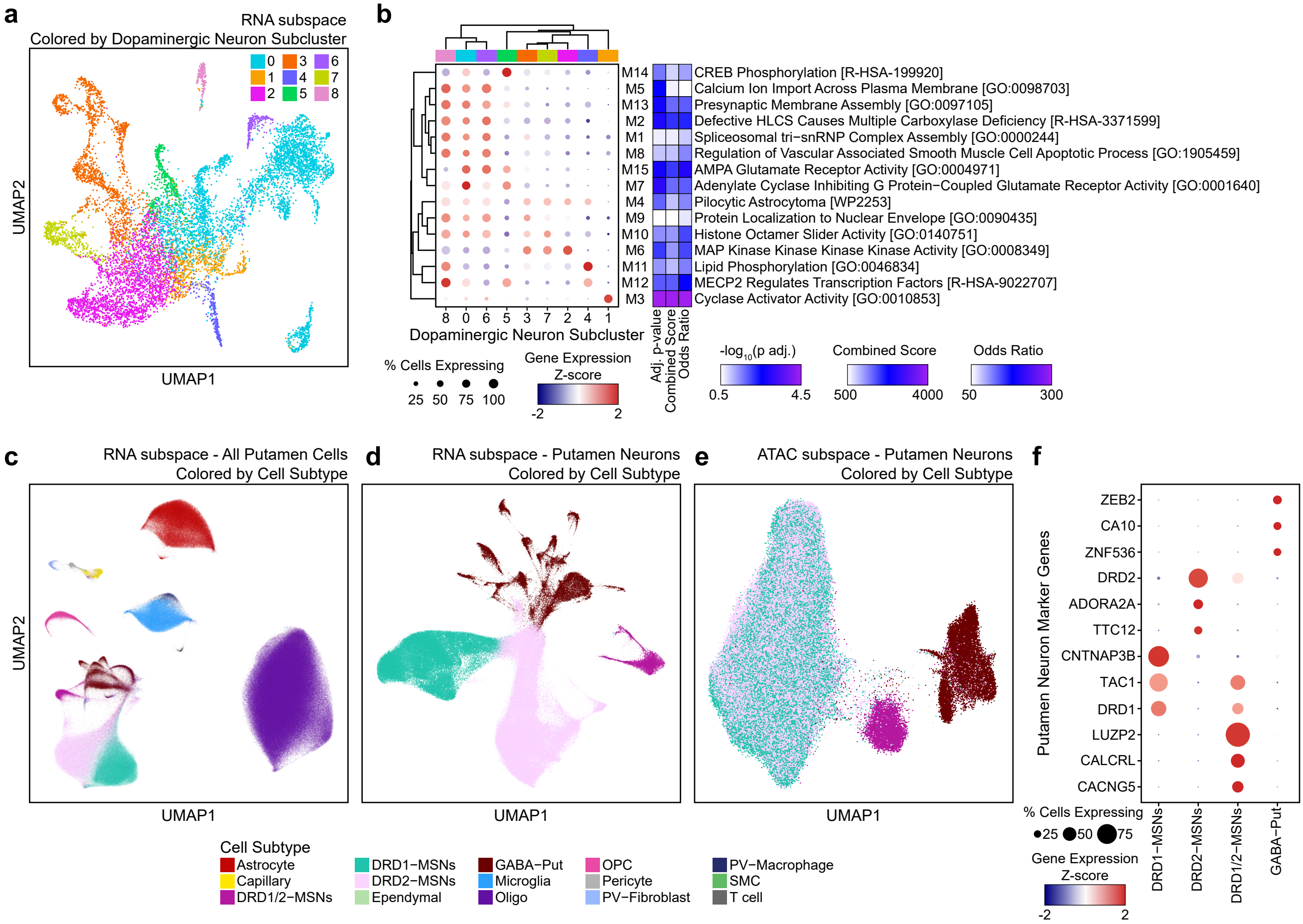

Supplementary Figure 4

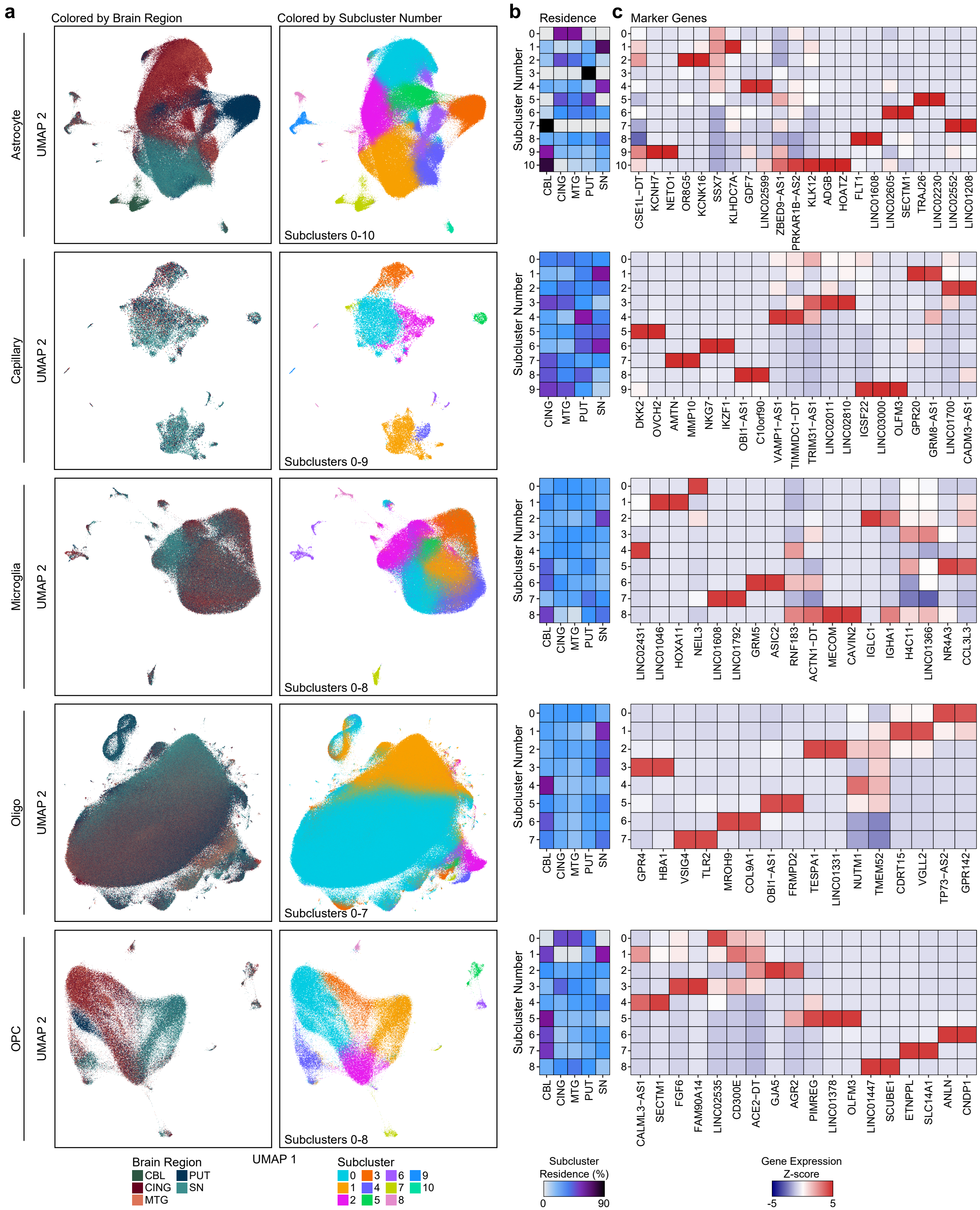

Supplementary Figure 5

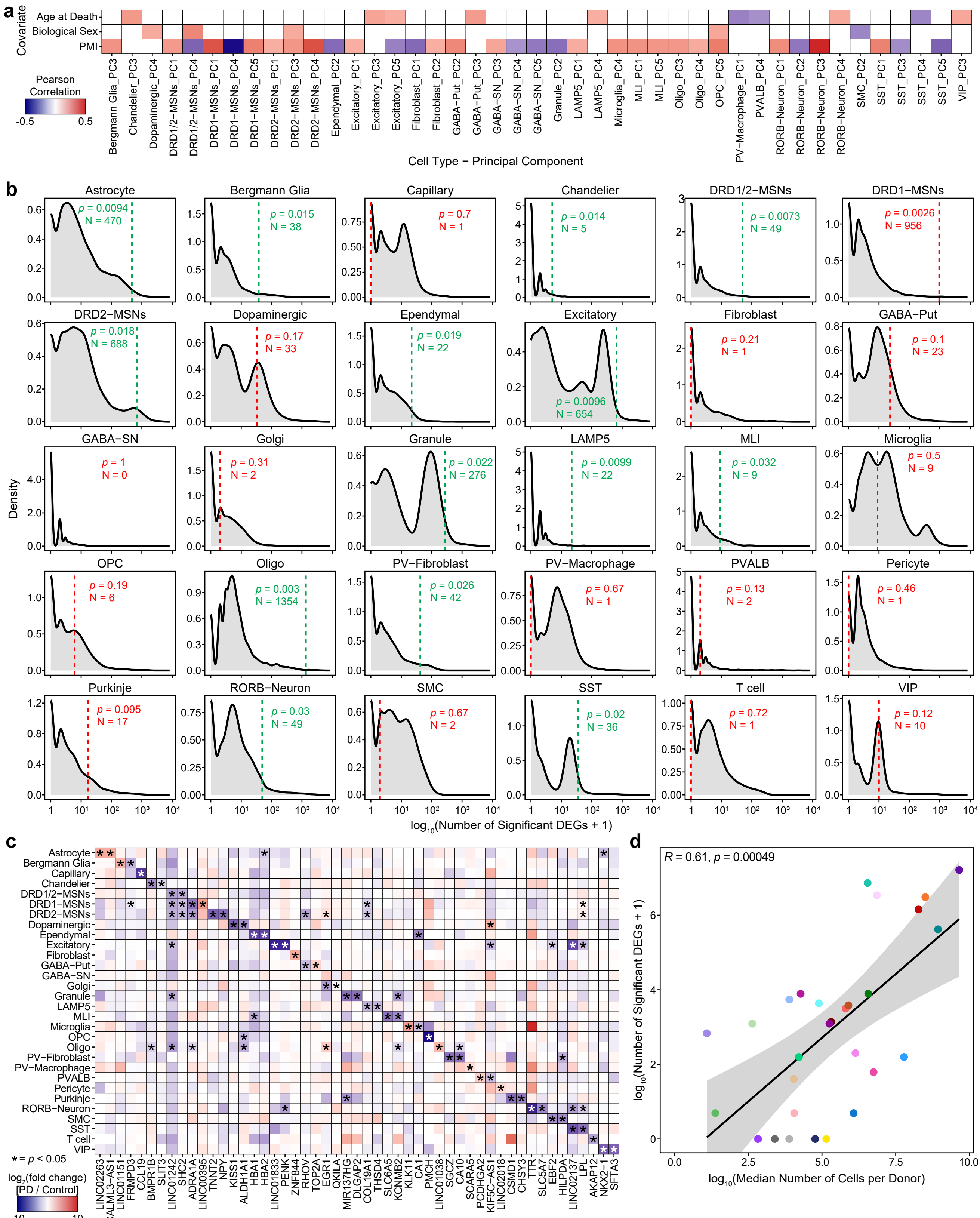

Supplementary Figure 6

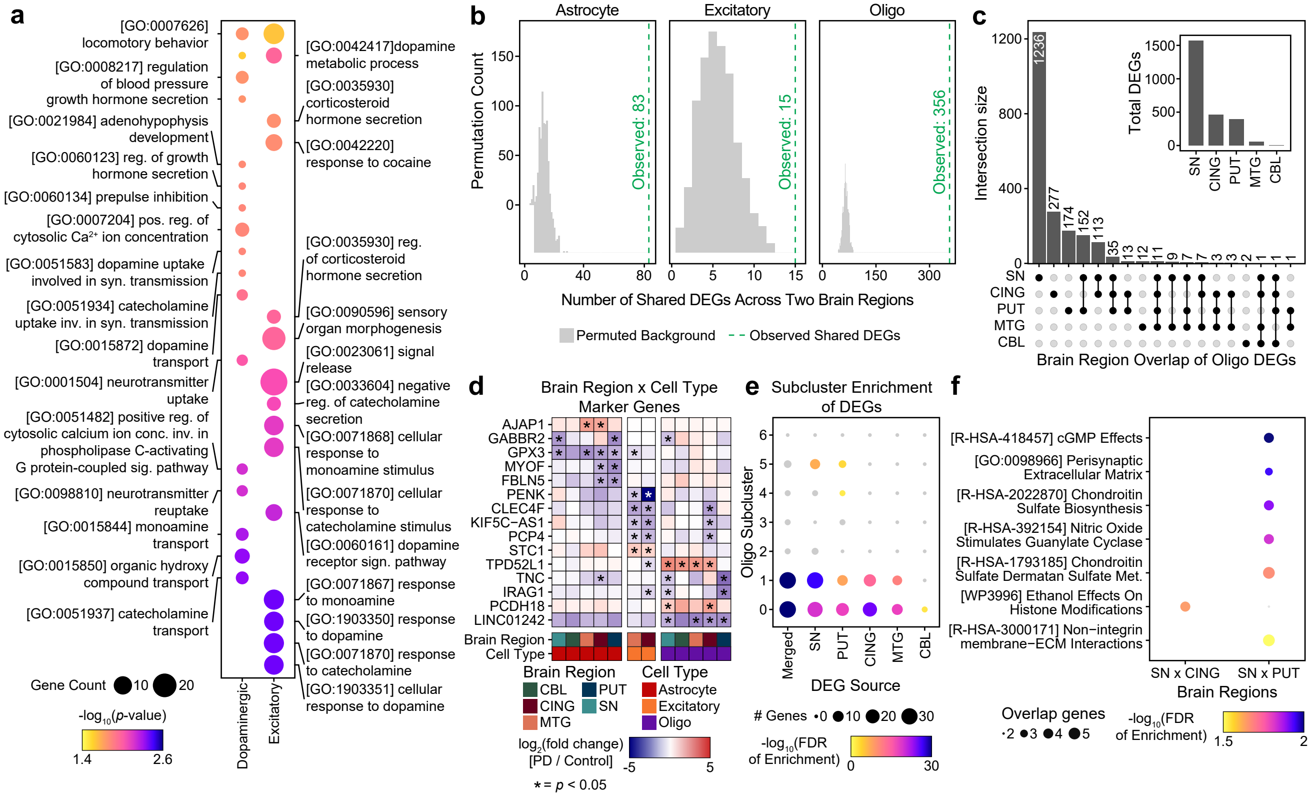

Supplementary Figure 7

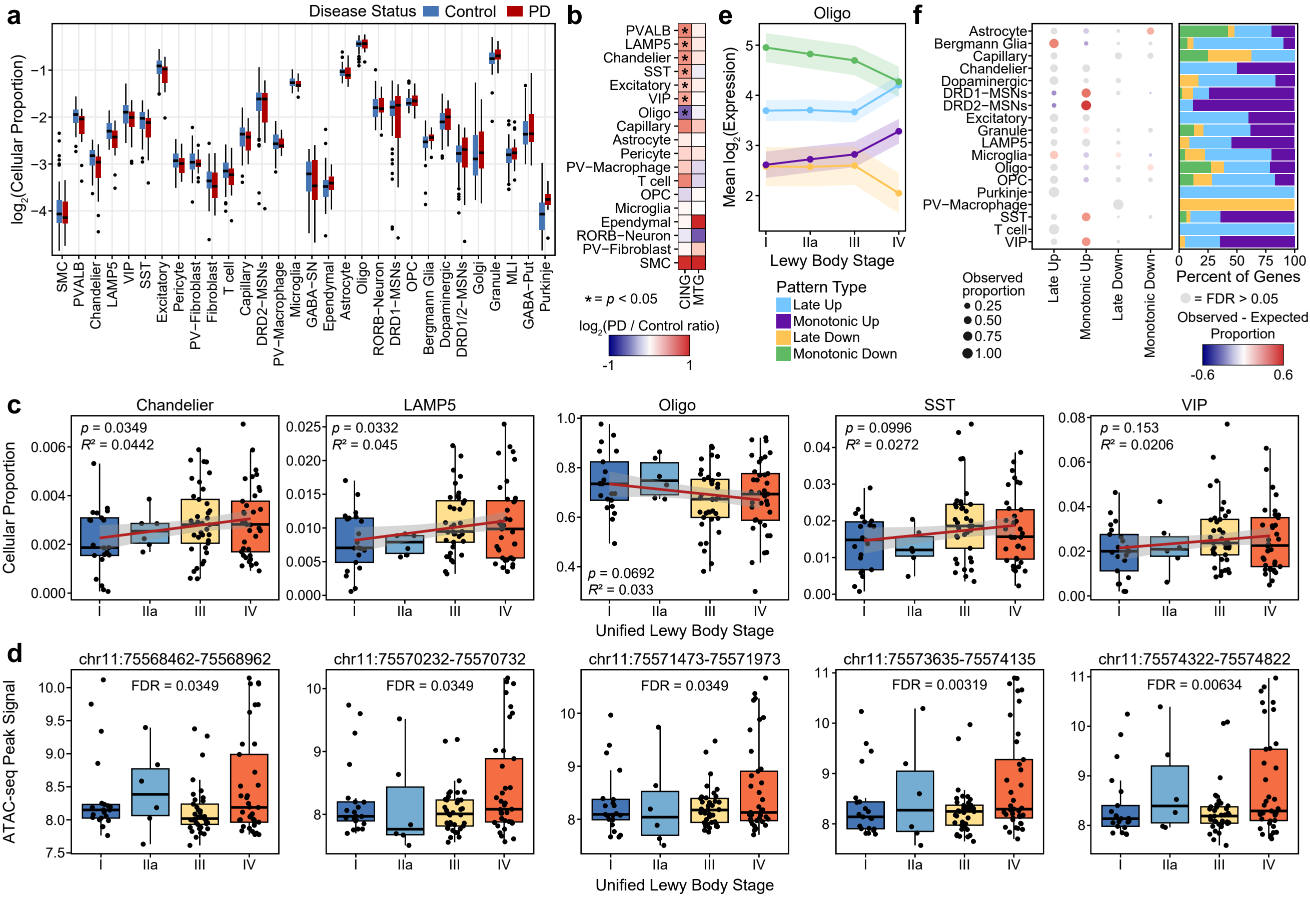

Supplementary Figure 8

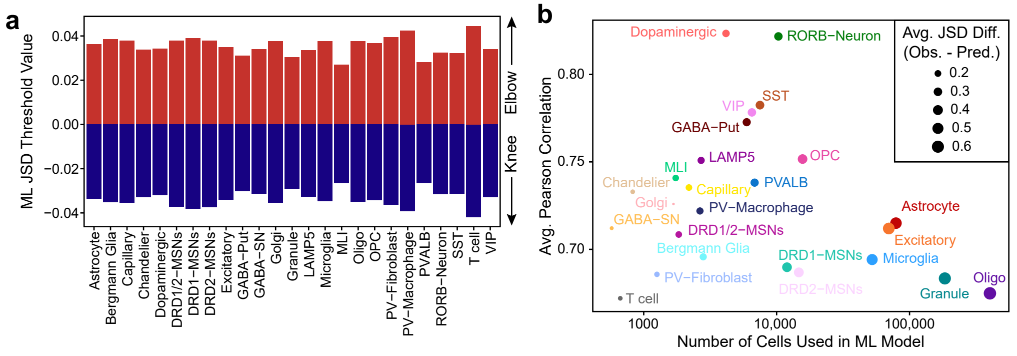

Supplementary Figure 9

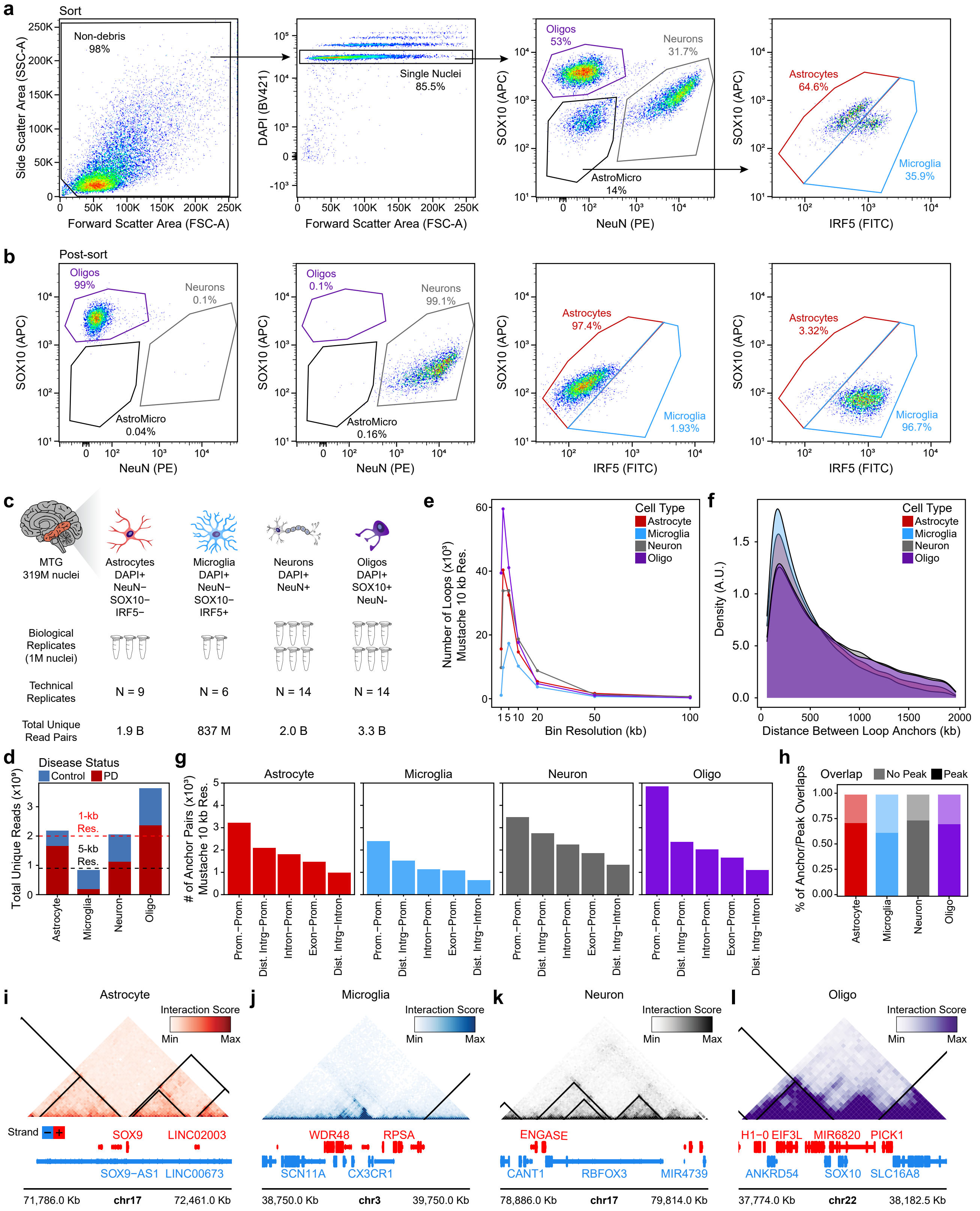

Supplementary Figure 10

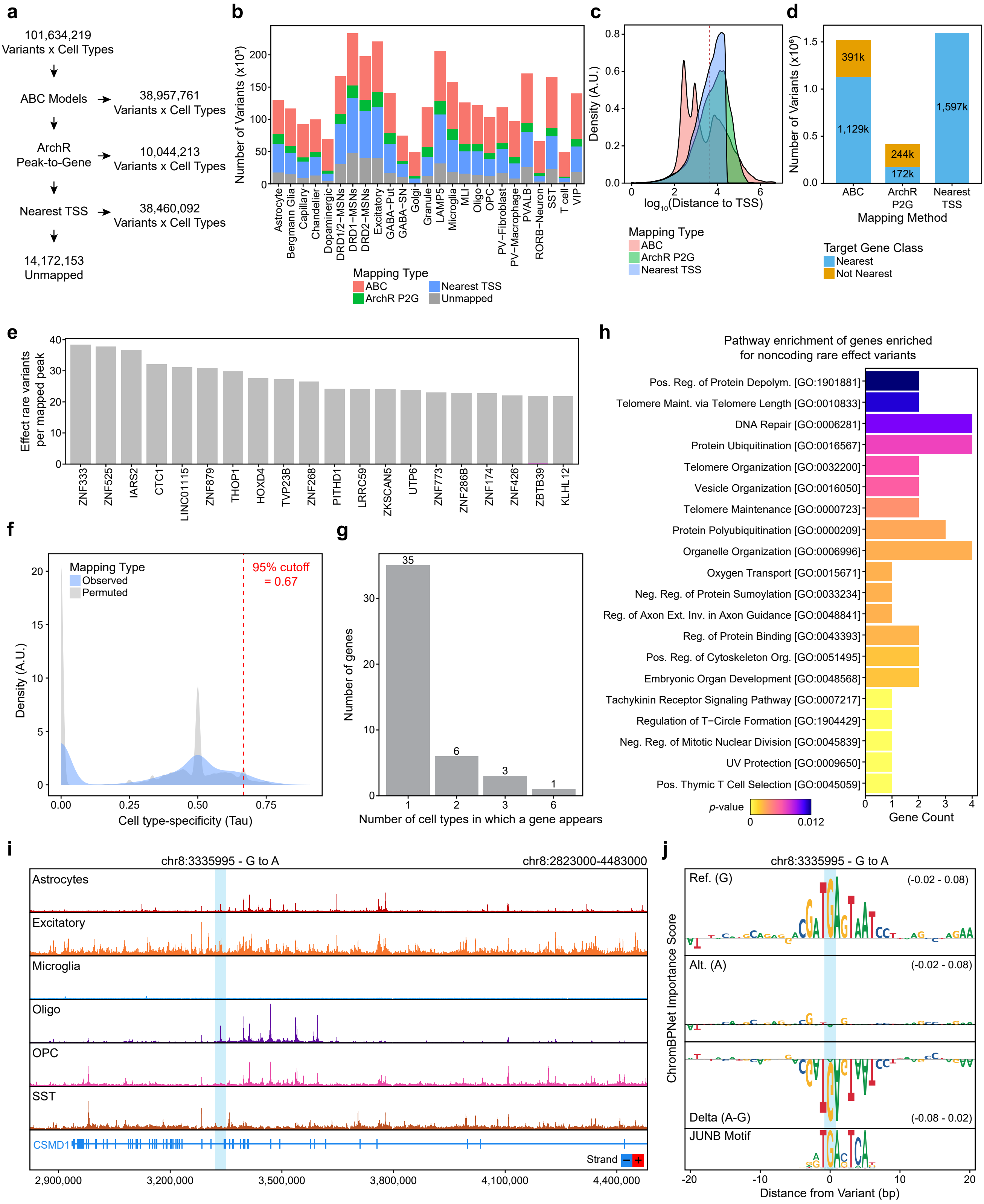

Supplementary Figure 11

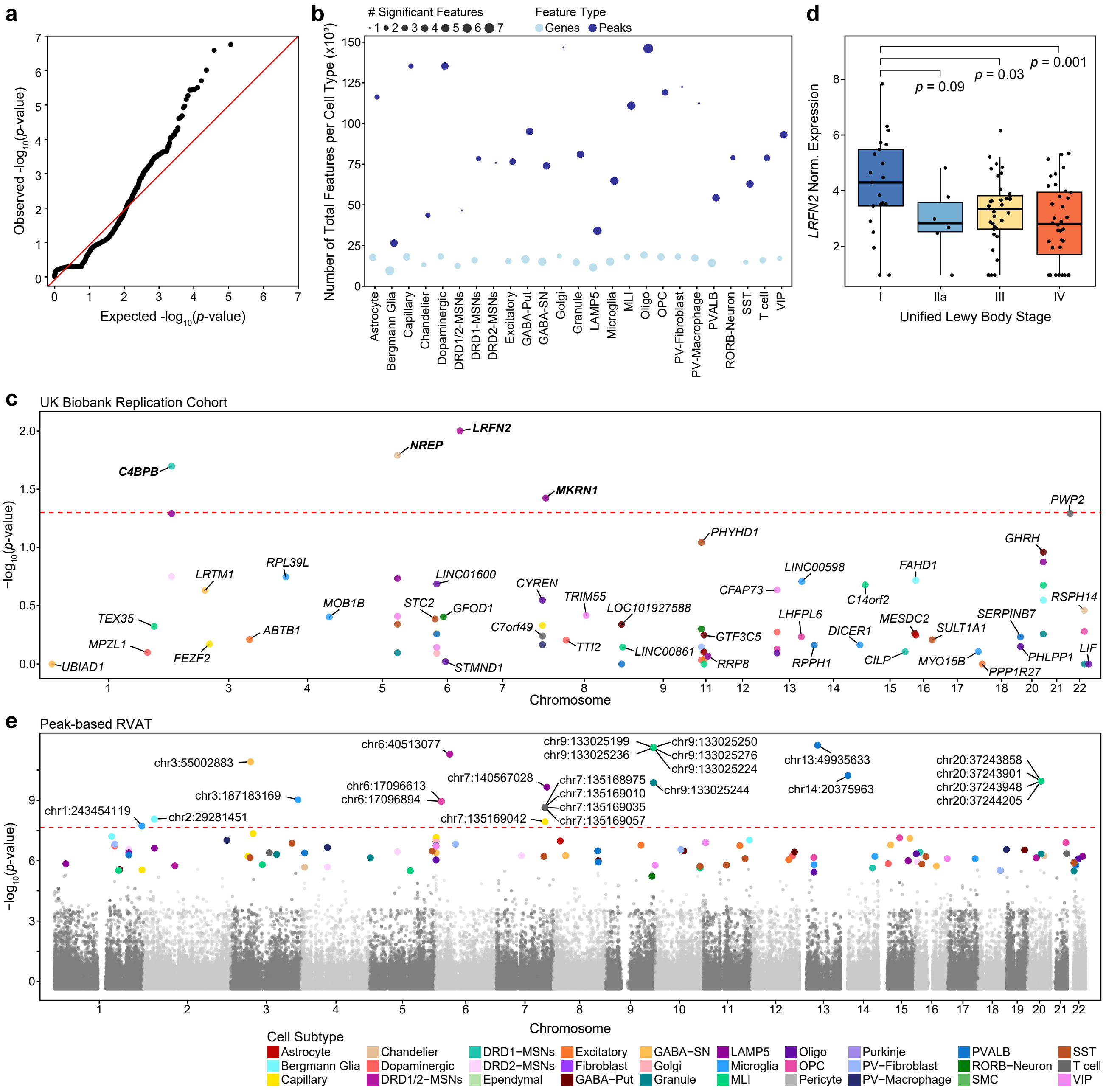

Supplementary Figure 12
